# Effects of a nonlocal microstructure on peeling of thin films

**DOI:** 10.1007/s11012-024-01786-2

**Published:** 2024-04-24

**Authors:** Riccardo Cavuoto, Luca Deseri, Massimiliano Fraldi

**Affiliations:** 1 Department of Structures for Engineering and Architecture, University of Naples “Federico II”, via Claudio 21, 80125 Naples, Italy; 2 Department of Neuroscience and Reproductive and Odontostomatological Sciences, University of Naples “Federico II”, via Pansini 5, 38131 Naples, Italy; 3 Department of Civil, Environmental and Mechanical Engineering, University of Trento, via Mesiano 77, 38123 Trento, Italy; 4 Department of Mechanical Engineering and Material Sciences, MEMS-SSoE, University of Pittsburgh, 3700, O’Hara Street, Pittsburgh, PA 15261 USA; 5 Department of Civil, Environmental Engineering, Carnegie Mellon University, Porter Hall 119, Pittsburgh, PA 15213-3890 USA; 6 Department of Mechanical Engineering, Carnegie Mellon University, 5000 Forbes Avenue, Pittsburgh, PA 15213 USA; 7 Laboratory of Integrated Mechanics and Imaging for Testing and Simulation (LIMITS), University of Naples “Federico II”, via Claudio 21, 80125 Naples, Italy; 8 Département de Physique, LPENS, École Normale Supérieure-PSL, 24 Rue Lhomond, 75005 Paris, France

**Keywords:** Peeling, Delamination, Peridynamics, Nonlinear mechanics

## Abstract

In this work, starting from an approach previously proposed by the Authors, we put forward an extension to the large deformation regime of the dimensionally-reduced formulation for peridynamic thin plates, including both hyperelasticity and fracture. In particular, the model, validated against numerical simulations, addresses the problem of the peeling in nonlocal thin films, which when attached to a soft substrate highlights how nonlocality of the peeled-off layer might greatly influence the whole structural response and induce some unforeseen mechanical behaviours that could be useful for engineering applications. Through a key benchmark example, we in fact demonstrate that de-localization of damage and less destructive failure modes take place, these effects suggesting the possibility of *ad hoc* conceiving specific networks of nonlocal interactions between material particles, corresponding to lattice-equivalent structure of the nonlocal model treated, of interest in designing new material systems and interfaces with enhanced toughness and adhesive properties.

## Introduction

In laminated composites, peeling refers to the separation or delamination of layers within the composite material. This phenomenon can significantly compromise the structural integrity and performance of the composite structure, especially that of laminated composites—structures made by bonding together layers of different materials. Peeling can weaken the bonds between these layers, leading to a loss of structural integrity, reducing the composite material’s mechanical properties, such as strength and stiffness, and accelerating the degradation of the material over time. Addressing this issue is especially critical in applications where the composite is subjected to extreme mechanical loads or stresses, and applications where the composite is designed for specific functional purposes, such as in aerospace or automotive components.

Peeling has therefore been extensively studied in the scientific literature [1–29]. A large amount of experimental and theoretical works explore the effects of several parameters, such as the peel angle, the thickness of the peeled-off material and the influence of the type of adhesion between the materials. Yet only a few studies focus on the effects of existing microstructures on the overall response [30–33].

Various numerical methods for fracture predictions have recently been developed to address the prediction of peeling forces and delamination paths, such as the cohesive-zone model (CZM) and the phase-field approach to fracture mechanics (PF) [34–42]. These models, though, suffers from some limitations, namely the a priori knowledge of crack path for the CZM and the physically unclear boundary condition for the phase-field approach.

Several of such limitations are circumvented by Peridynamics, a relatively recent theory introduced by Silling in [43, 44]. Peridynamics is a strongly nonlocal theory that has several advantages over other approaches when dealing with damage and fracture transition, but also with crack propagation. These advantages are both related to the natural correlation between fracture and nonlocal fields [45], as well as to the intrinsically direct way to implement damage at the constitutive level in the peridynamic theory [46].

In the peridynamic literature, models for thin bodies can be divided into two main categories: (i) fully three-dimensional models [47–49], and (ii) dimensionally-reduced models [44, 50–57]—namely 2D. While the former approach boasts numerous works [58, 59], among which many deal with delamination, fewer dimensionally-reduced models have been put forward and only one of them treats delamination explicitly [60]. The advantages of a dimensionally reduced formulation in bond-based peridynamic theory are numerous: (i) the dimensional reduction drastically lowers the computational effort which is a serious issue in peridynamics computational simulations, where the number of elements grows exponentially with the number of nodes in the model; (ii) the reduced formulation foster a physical interpretation of the various terms emerging from the reduction procedure giving a better insight on the phenomena related to nonlocal material of the peridynamic kind.

In [60], the Authors have witnessed unusual properties of nonlocal peridynamic thin structure emerging from the results of the dimensionally reduced formulation; properties that, thanks to a well-established equivalence between the peridynamic bond-based theory and a particular network of trusses, can be ascribed to the nonlocal character of this microstructure.

In the present work, we operate towards expanding the reduced formulation discussed [60] to the large displacement regime and test the model in a very specific peeling test against numerical simulations. It is found that the nonlocal character of peridynamics leads to interesting properties, such as de-localization of damage and changes in the force required to induce peeling in a thin film.

The paper is organised as follows: in Sect. 2 a brief introduction of bond-based peridynamics is presented, while the nonlinear regime is implemented in the constitutive equations (Sect. 2.1). In Sect. 3 the reduced formulation is presented and extended to the nonlinear deformations case in a computational fashion, where the elastic problem is set up and a corresponding solution algorithm is presented. In Sect. 4 the model is applied, and compared with numerical solutions, to study the peeling of a thin nonlocal film from a soft substrate. A sensitivity analysis of horizon size is also performed. Lastly, in Sect. 5, a discussion about the results obtained is carried out and implications of the findings are presented.

## Fundamentals on nonlinear bond-based peridynamics

**Fig. 1:**
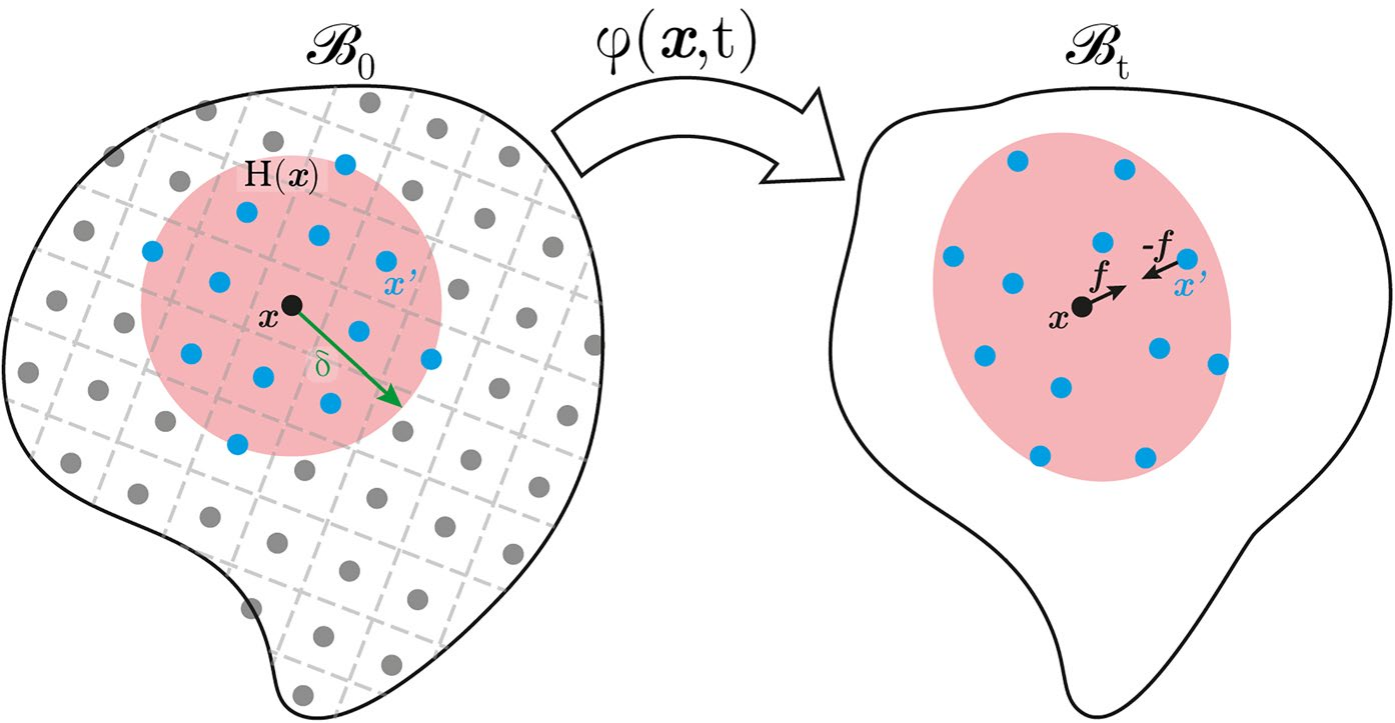
Mapping of the undeformed peridynamic body into its deformed state, corresponding to some step t of a loading process. Material particle $\varvec{x}$ interacts with all the ones belonging to a certain region centred in $\varvec{x}$ (in red), called its *family*, $\mathbb {H}(\varvec{x})$. Interactions are made explicit through the rise of reciprocal forces exerted on pairs $(\varvec{x},\varvec{x}')$ due to deformation

Bond-based peridynamics [43] is a strongly nonlocal continuum theory that models a body $\mathcal {B}$ as a collection of particles, see Fig.1.

Static equilibrium of the body under exam is imposed according to the following integral equation:

1 $$\begin{aligned} \int _\mathbb {H} \varvec{f}(\varvec{x},\varvec{x}', \varvec{u}(\varvec{x}),\varvec{u}(\varvec{x}'))\textrm{d}\varvec{x}'+\varvec{b}(\varvec{x})=\varvec{0}, \end{aligned}$$

where $\varvec{u}$ is the displacement field mapping, through a function $\varphi (\varvec{x},t)$ with no requirements of regularity, the body under exam onto its deformed configuration, $\varvec{b}$ is the vector of distributed loads and $\varvec{f}$, called the pairwise force function, is the vector representing the interaction among each pair of particles. These forces not only represent nearest and next-to-nearest neighbour interactions but more generally forces between each point $\varvec{x}$ and all the others $\varvec{x}'$ *close enough* to it (see Fig. 1, points in blue). In other words, such points $\varvec{x}'$ belong to $\mathbb {H}(\varvec{x}) = \{\varvec{x}'\ s.t.\ |\varvec{x}'-\varvec{x}|\le \delta \}$, a neighbourhood of $\varvec{x}$, where $\delta$ is called *horizon* [43, 60, 61]. Typically, in the elastic regime a linear relationship between $\varvec{f}$ and a generalised measure of strain, *s*, is assumed,

2 $$\begin{aligned} \varvec{f}\left( \varvec{x},\varvec{x}', \varvec{u}(\varvec{x}),\varvec{u}\left( \varvec{x}'\right) \right) =c\mu \ s\left( \varvec{x},\varvec{x}'\right) \ \varvec{e}\left( \varvec{x},\varvec{x}'\right) , \end{aligned}$$

where *c* is a constant—called the bond constant—while $\varvec{e}$ is the unit vector expressing the direction of the bond. Finally, $\mu$ is a history-dependent scalar-valued function, called failure parameter, which enforces bond breakage under tension only [60, 62]. Such a parameter is equal to 1 until the bond is intact, while it takes value zero whenever a critical elongation $s_{cr }$ (or critical energy $\omega _{cr }$) is reached [63–67]. Hereon, a more compact notation is used, namely $\varvec{\xi }=\varvec{x}'-\varvec{x}$, $\varvec{\eta }=\varvec{u}'-\varvec{u}$ (where $\varvec{u}'=\varvec{u}(\varvec{x}')$) and $\lambda =|\varvec{\xi }+\varvec{\eta }|/|\varvec{\xi }|$ for the stretch, which allows to write $\varvec{e}=(\varvec{\xi }+\varvec{\eta })/|\varvec{\xi }+\varvec{\eta }|$.

If the force function admits a potential, then a pairwise potential function can be defined as follows:

3 $$\begin{aligned} \omega (\varvec{\xi },\varvec{\eta })= \int \varvec{f}(\varvec{\xi },\varvec{\eta })\cdot \textrm{d}\varvec{\eta } = c\ |\varvec{\xi }|\int \mu \ s(\lambda )\textrm{d}\lambda \quad . \end{aligned}$$

To compare the modelling capacity of the peridynamic approach with that of standard local continua, the bond constant *c* is typically calibrated by enforcing an energetic comparison [68]. A very consistent strategy is the one adopted in [69], where the equivalence between the nonlocal peridynamic energy density and the standard local elastic one is assumed under the hypothesis of small horizons and small displacements. This type of calibration leads to writing the following relationship between *c* and the Young’s modulus, E, of an equivalent homogeneous linear elastic local solid:

4 $$\begin{aligned} c = \frac{9E}{\pi \delta ^3}, \end{aligned}$$

written for the purely 2D case.

### Nonlinear regime formulation

We hereby briefly summarise the concepts and measures of a large deformation theory applied to bond-based Peridynamics, as some of the following relations are already present in the relevant literature [55, 70–77]. Nevertheless, it is detected that some quantities related to the peridynamic solid have not been explicitly quantified in this context.

In trying to compensate this drawback, it is useful to recall that the microstructural interpretation of a bond-based peridynamic solid is that of a network of trusses that connect the material points in pairs and exchange collinear forces [43, 60, 64, 69, 78–80]. To account for the large deformation of these trusses a nonlinear measure of deformation must be considered. This is because the conventional strain measure

$$s=\lambda -1,$$

fails at remaining consistent in that regime. Hence, a more adequate strain measure for the nonlinear geometric regime turns out to be the following:

5 $$\begin{aligned} s=\frac{\log \lambda }{\lambda }. \end{aligned}$$

The complete derivation of Eq. (5) is shown in the Appendix. According to (5) the resulting pairwise force function and pairwise potential function are:

6 $$\begin{aligned} \varvec{f}&=c \mu \ \frac{\log \lambda }{\lambda } \frac{\varvec{\xi }+\varvec{\eta }}{|\varvec{\xi }+\varvec{\eta }|},\\ \omega &=\frac{1}{2}c \mu |\varvec{\xi }|(\log \lambda )^2+(1-\mu )\omega _{cr }. \end{aligned}$$

## Thin plates peridynamic model accounting for delamination

**Fig. 2:**
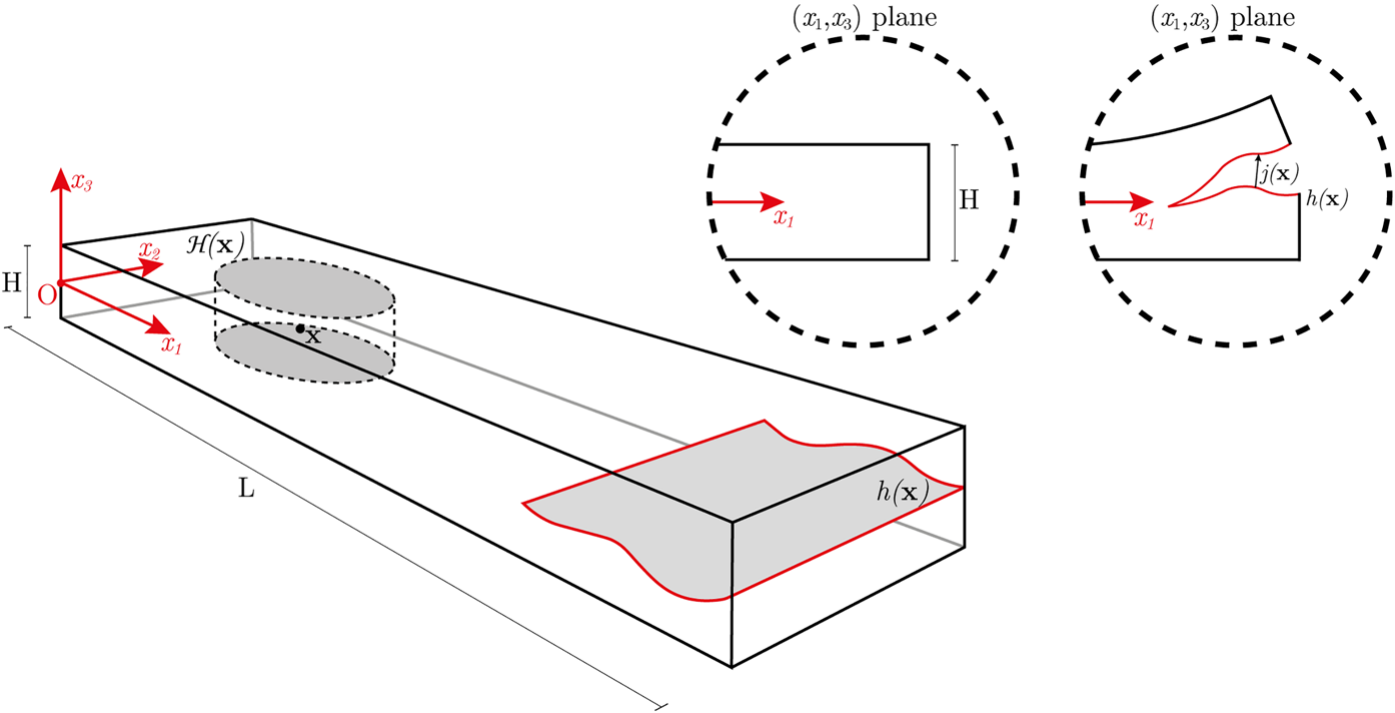
Geometric schematic for the thin solids under exam (on the left) and for the class of admissible displacement fields (on the upper right). A delamination surface $h(\varvec{\textrm{x}})$ is allowed to propagate through the thickness of the solid, separating the two portions of the system by a differential $\varvec{j}(\varvec{\textrm{x}})$

In a previous work [60] a dimensionally reduced formulation for the through-thickness delamination of thin plates has been introduced. The formulation relies on an additive decomposition of the displacement field, $\varvec{u}(\varvec{x})$, of thin solids into an *absolutely continuous* part, $\varvec{u}_{\alpha }(\varvec{x})$, and a jump part, $\varvec{u}_J(\varvec{x})$, see Fig. 2. There, the formulation has been used to explore the effects of a nonlocal microstructure at the onset and early-stage development of crack propagation. The obtained results showed several unconventional properties, such as distal nucleation of the delamination surface for the most usual peeling tests and asymmetrical propagation for the effects induced by couples. The possibility of predicting damage away from the loaded zones is a very promising outcome for several engineering applications related to the development of prototypes.

**Fig. 3:**
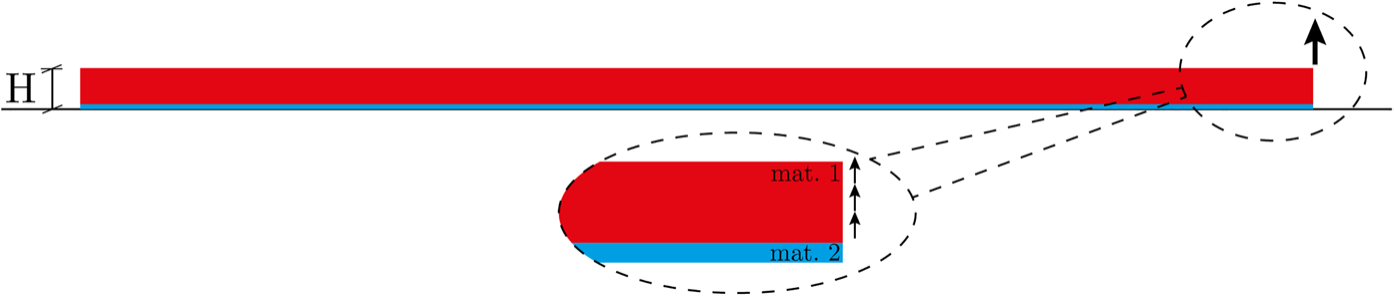
Representative sketch of the geometrical conditions under exam for peeling of an elastic thin film (in red), denoted as Material 1, on a deformable substrate (in blue), denoted as Material 2. In the sequel, Material 2 will be modelled with a standard local continuum theory to reproduce its local behaviour. Material 1, on the contrary, is considered to be either local or nonlocal, and the effects of a more pronounced nonlocality on the mechanical behaviour of the composite are explored. Throughout the work perfect adherence is assumed for the interface between Materials 1 and 2

Given the thin element’s geometrical features, the absolute continuous part of the displacement was approximated by a through-thickness series expansion: $\varvec{u}_{\alpha }=\mathcal {A}(x_1,x_2)+\mathcal {B}(x_1,x_2) x_3+\cdots$. In contrast, for the jump part, a relatively general ansatz is found in [60] and reads as follows: $\varvec{u}_J(\varvec{x})=\varvec{j}(x_1,x_2)\ H(x_3-h(x_1,x_2))$, where *H* is the Heaviside function, $x_i$ are the main axes of a Cartesian reference system centred on the mid-plane of the plate with $x_3$ being the through-thickness direction. Lastly, $h(x_1,x_2)$ and $\varvec{j}(x_1,x_2)$ are functions representing the delamination surface and jump quantity, respectively. These quantities are all explicitly displayed in Fig. 2. As is clearly stated in [60], such a displacement ansatz limits the delamination surface to being unique therefore only one surface can nucleate and propagate within the thickness, which is quite reasonable for thin objects. This assumption which may have had a certain influence in the study of homogeneous thin plates treated in [60], looses much of its limitations when a well-defined preexisting horizontal interface exists such as in the present work (see e.g. Fig. 3).

### Delamination of a thin film on a substrate

The scope of the present paper is to present an extension of the above-mentioned model to the large displacement regime. This extension permits one to follow the evolution of damage until the collapse of specimens, even when large rotations and translations do occur before the loss of integrity of the system. This allows for investigating the differences in terms of the mechanical response of nonlocal architectured thin solids when compared with standard ones behaving locally in such large displacement regimes.

Similarly to what was performed in [60], from the energetic point of view, one can write the total Lagrangian as the difference between work expended by external loads, $\mathcal {W}$, and the strain energy of the nonlocal continuum, $\mathcal {E}$:

7 $$\begin{aligned} \mathcal {L}{} & {} =\mathcal {W}-\mathcal {E}=\int \limits _{\mathcal {B}}\left( p- \int \limits _{\mathcal {B}'} \omega \ \textrm{d}V' \right) \,\textrm{d}V\nonumber \\{} & {} = \int \limits _{\mathcal {B}_0}\left( \overline{p}-\int \limits _{\mathcal {B}_0'} \overline{\omega }\ \textrm{d}V_0' \right)\, \textrm{d}V_0\quad , \end{aligned}$$

where $\mathcal {B}$ stands for the current configuration of the body, $\mathcal {B}_0$ is the reference one, $\omega$ is that of Eq. (6), $p=\varvec{b}\cdot \varvec{u}$ and their overbar version is the result of the pullback of the integrals onto the reference configuration [81]. Therefore,

8 $$\begin{aligned} \mathcal {L}=\int \limits _{S}\left[ \int \overline{p}\ \textrm{d}x_3-\int \limits _{S'} \left( \frac{1}{2}\int \int \overline{\omega }\ \textrm{d}x'_3\textrm{d}x_3 \right) \textrm{d}S'\right] \textrm{d}S, \end{aligned}$$

where *S* and $S'$ are regions defined by the reduction plane, i.e. the plane with respect to which the dimensional reduction is performed, while $x_3$ is the normal direction to the plane of dimensional reduction. See [60] for a thorough derivation.

Equilibrium configurations are then searched for by solving

9 $$\begin{aligned} \delta \mathcal {L}[\varvec{u}]=\int \limits _S \left[ \frac{\partial p_{red }}{\partial \varvec{u}} - \int \limits _{S'} 2 \frac{\partial \omega _{red }}{\partial \varvec{u}}\textrm{d}S'\right] \cdot \delta \varvec{u}\ \textrm{d}S =0, \end{aligned}$$

with $\omega _{red }$ and $p_{red }$ being the result of the through-thickness integrations in (8). Since the displacement field, $\varvec{u}$, is itself a function of various scalar functions, specified by the ansatz mentioned above, Eq. (9) is actually a system of equations, which can be symbolically expressed by replacing the derivation variable $\varvec{u}$ with $\mathcal {A},\mathcal {B},\cdots ,\varvec{j},h$.

#### Explicit nonlinear formulation and step-by-step solution scheme

 A direct way to deduce a representation formula for the symbolic expression of the reduced form of the peridynamic pairwise potential function would be quite challenging to conceive and it goes beyond the scope of this paper. Therefore, a computational procedure is devised to solve (9) step-by-step along the loading process. At the generic loading step, the nonlinear pairwise potential function is approximated by the following quadratic form

10 $$\begin{aligned} \omega{} & {} \approx \mu [\omega (\varvec{\xi },\varvec{\eta })+\varvec{f}(\varvec{\xi },\varvec{\eta })\cdot \Delta \varvec{\eta }+\Delta \varvec{\eta }\cdot \mathbb {C}(\varvec{\xi },\varvec{\eta })\Delta \varvec{\eta }/2]\nonumber \\{} & {} \quad +(1-\mu )\omega _{cr }\, \end{aligned}$$

where the unknowns $\Delta \varvec{\eta }$, the increment in the displacements, are the only varying quantities since all the others are being held fixed, and $\mathbb {C}$ the micro-modulus function representing the tangential stiffness is defined as

11 $$\begin{aligned} \mathbb {C}(\varvec{\xi },\varvec{\eta })=\frac{c}{|\varvec{\xi }|}\ \left( \frac{\log \lambda }{\lambda ^2}\ \varvec{I}+\frac{1-2\log \lambda }{\lambda ^2}\ \varvec{e} \otimes \varvec{e}\right) , \end{aligned}$$

where $I_{ij}=\delta _{ij}$, $\delta _{ij}$ is the Kronecker delta, and $\lambda$ is the stretch of the bond. Functions $\omega (\varvec{\xi },\varvec{\eta })$ and $\varvec{f}(\varvec{\xi },\varvec{\eta })$ of (6), are being evaluated at the specific increment.

System (9) can then be rewritten in the new unknowns, which are the incremental displacements, and solved using a Ritz–Galerkin approach.

At each increment of the loading process, the reduced energy can be evaluated by assuming a perfectly elastic behaviour, a hypothesis that becomes more realistic when the increments are small.

At the end of each step, a check on bond elongation is performed and those that are found to be above the critical threshold are removed from the model by associating a value of $\mu =0$. These steps are summarised in Algorithm 1.

**Algorithm 1:**
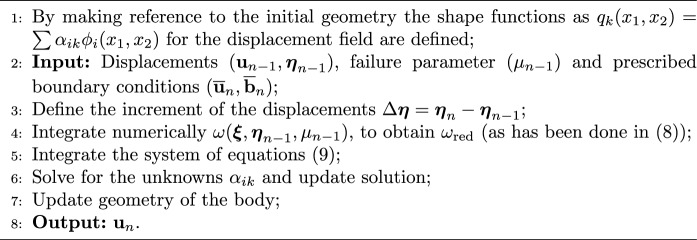
Scheme for the solution of (9) at a step $n \ge 1$

## Peeling of a thin peridynamic film on deformable substrate

We here apply the nonlinear and computationally reduced model for the delamination of peridynamic thin plates to study the peeling-off behaviour of a flexible film attached to a soft substrate (see Fig. 3).

In detail, two layers of different homogeneous materials attached one on top of the other, with a flat and perfect interface parallel to the mid-plane of each material, are subjected to a displacement-induced peeling test. The two layers share the same stiffness and strength response. The substrate, clamped to the ground (null relative displacements and rotations) is isotropic and homogeneous, exhibiting a local response. The upper layer, Material 1 in Fig. 3, on the contrary, is inherently nonlocal (peridynamic), and thus equivalently described by a lattice with an intricate network of trusses [60].

The effects of a varying horizon, $\delta$, on the peeled film—the upper layer—are explored.

The various cases are compared in terms of crack nucleation and propagation path under the condition of identical bulk stiffness and strength properties. The stiffness of the bulk portion of the body is calibrated by using (4), while the strength is determined by utilising a relationship between critical bond elongation $s_{cr}$ and critical surface energy for mode-I fracture propagation in standard local continua. To do so, the energy required to create a new surface in the bulk region of a bond-based peridynamic solid is computed. To this end, by following the ideas in [44, 46], and by equating the peridynamic energy required to form a new surface under isotropic expansion to the critical energy required to open a new surface in a local solid, $\mathcal {G}$, one gets:

12 $$\begin{aligned} \mathcal {G}{} & {} =2\int _{0}^{\delta }\int _z^{\delta }\int _{0}^{\cos ^{-1}z/|\varvec{\xi }|} \omega \ |\varvec{\xi }|\ \textrm{d}\theta \ \textrm{d}|\varvec{\xi }|\ \textrm{d}z \nonumber \\{} & {} = \frac{1}{4} c \delta ^4\ \log (1+s_{cr})^2, \end{aligned}$$

where $\omega$ is that of equation (6) assuming $\mu =1$ (see e.g. [46] chapter 6 Eqs. (6.11–6.19)), which then solved for $s_{cr}$ gives:

13 $$\begin{aligned} s_{cr}=e^{\sqrt{4\mathcal {G}/(c\ \delta ^4)}}-1 = e^{\sqrt{4\pi \mathcal {G}/(9E\ \delta )}} -1, \end{aligned}$$

where the last equality is in virtue of Eq. (4).

The reduced formulation is also compared with full-scale bond-based peridynamic analysis performed through ANSYS^®^. Following established considerations available in [43, 47, 60], the correct way to discretise a bond-based peridynamic problem is using a grid of nodes. In particular, for a pair of nodes (*i*, *j*) each of them is associated with its own small neighbouring volume of peridynamic material, say $V_i$ for the node *i*. For pairs of nodes (*i*, *j*) included in the horizon (i.e. satisfying a relative distance threshold, represented by the horizon) they are connected by truss elements whose stiffness, $k_{ij}$, amounts to $2k_{ij}=c V_i V_j /|\varvec{\xi }|$.

In the sequel, a benchmark result is presented for the type of test depicted in Fig. 3, showing the propagation of a crack in a local continuum characterised by a specific damage criterion. The benchmark is compared with the result of an analysis of the same test carried out on an equivalent lattice of trusses, which in [60] is shown to be a local limit of the peridynamic bond-based model.

### Lattice equivalent local continuum: a benchmark model

**Fig. 4:**
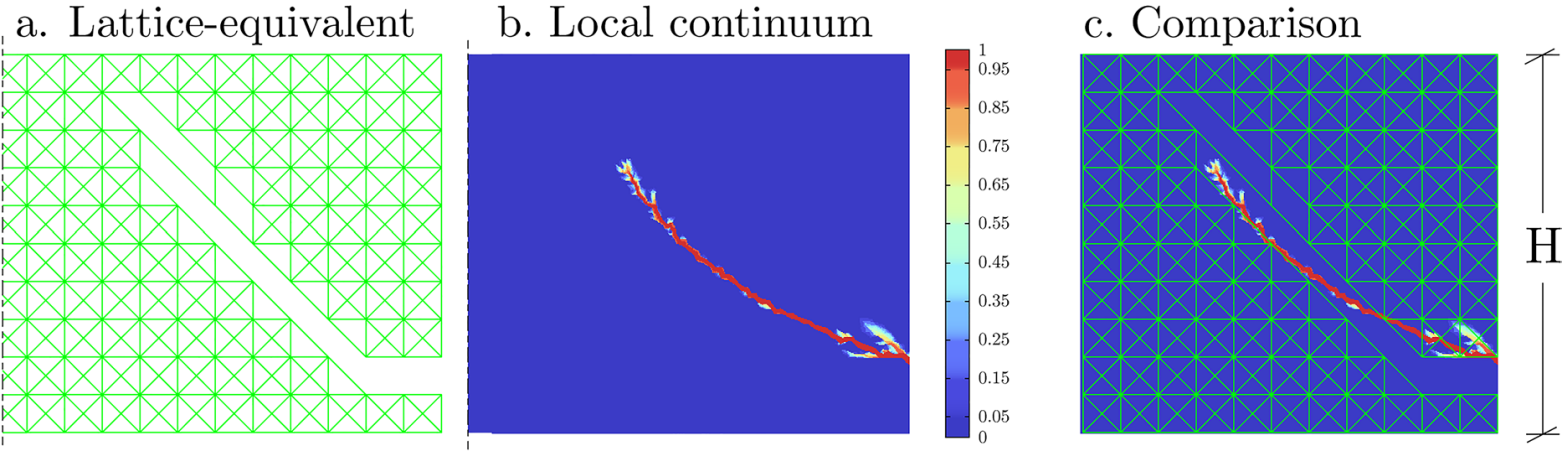
Frame of the loaded edge of a homogeneous local thin film loaded as shown in Fig. 4 and predictions of damage propagation according to a lattice equivalent model (**a**) and standard fracture mechanics model (**b**). Part (**c**) shows a direct comparison obtained by the overlapping of images (**a**) and (**b**)

 In the following, a benchmark analysis is portrayed, see Fig. 4. The homogeneous material under study is characterised by a local linear elastic and isotropic constitutive response $(E=1800MPa , \nu = 0.25)$ in a geometrically nonlinear regime with the Hencky strain measure. A scalar damage theory, relying on Rankine strain, with exponential strain softening [82], a tensile strength of $\sigma _{ts}=100$ MPa and a fracture energy per area of $G=10$ N/m is implemented for the post-elastic behaviour, to reproduce crack propagation in the solid. The boundary conditions of the test are those of Fig. 4, where the right edge of the specimen is partially pulled upwards, while the lower edge is held fixed.

The result of the local analysis (part b of Fig. 4) is compared with that of a coarse equivalent lattice, which well approximates a peridynamic bond-based continuum in the local limit [60] (see part (a) of the figure). A comparison of the two results is shown in (c) by superposition of the final stages of the analyses.

### Pronounced nonlocality

**Fig. 5:**
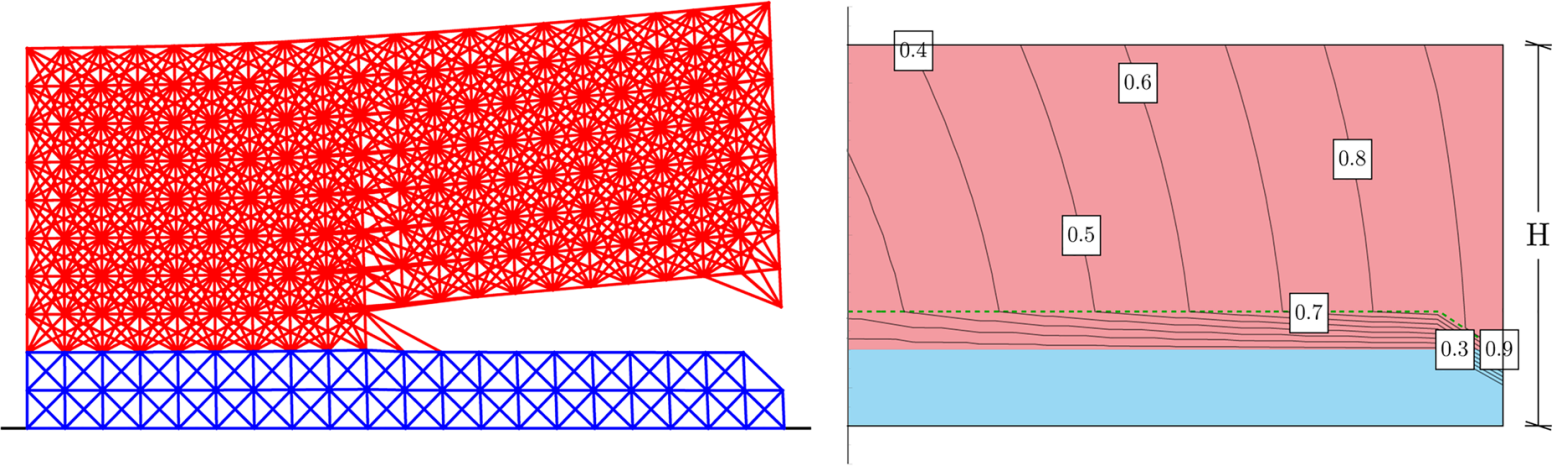
On the left: in red the layer of material which is being pulled upwards in a displacement-induced peeling test, while in blue a thin compliant substrate. The upper layer has a pronounced nonlocal microstructure ($\delta =0.3H$), while the lower layer is assumed to behave almost locally. The picture shows the intact bonds after an imposed vertical displacement of $u=40\%$H. On the right: map of the displacements showing contour lines normalised with respect to the peak imposed displacement of $u=40\%$H. In red and blue the thin layer and substrate respectively have the same overall stiffness and strength

 This paragraph presents the results of the displacement-induced peeling test carried out in the case of Material 1 having a pronounced nonlocal microstructure, i.e. bonds connect material particles that are at most at a distance of $\delta =0.3H$ from each other, see Fig. 5. The lattice parameters, such as the bond constant, *c*, and the bond critical stretch, $s_{cr }$, have been calibrated making use of Eqs. (4) and (13). Those allow for obtaining stiffness and strength of the bulk portion of the body which are comparable to those of the substrate. Calibration is performed as explained in the first part of Sect. 4.

**Fig. 6:**
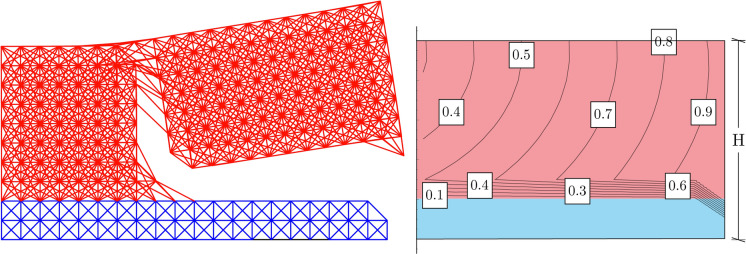
The final configuration (on the left) and contour plot of the displacements (on the right) for the analysis conducted using ANSYS^®^ on a thin film (in red, with $\delta =0.3H$) laying on a soft substrate (in blue) subjected to a peeling test and characterised by a microstructure which corresponds to the discrete approximation of bond-based peridynamics. The model is full-dimensional, meaning no reduction through the thickness has been performed. Final displacement of the test amounts to $u=45\%$H. Still, comparison with results from the reduced formulation shows a very close (qualitative and quantitative) resemblance

The results of the analysis on the nonlocal structure show a horizontal propagation of the crack tip which easily spans a length greater than the element thickness, only to propagate in the upper material at a distal section from the loaded end. These observations are reported in Fig. 5, where the final stage of the displacement-induced peeling test has been shown for the case of the dimensionally reduced peridynamic formulation. This result is confirmed by full dimensional analysis, carried out by using ANSYS^®^ software of the nonlocal lattice equivalent to the discrete approximation of bond-based peridynamics, as depicted in Fig. 6. It is worth highlighting that the two methodologies gave no differences in terms of fracture onset, both cases reproducing damage onset at the same loci.

**Fig. 7:**
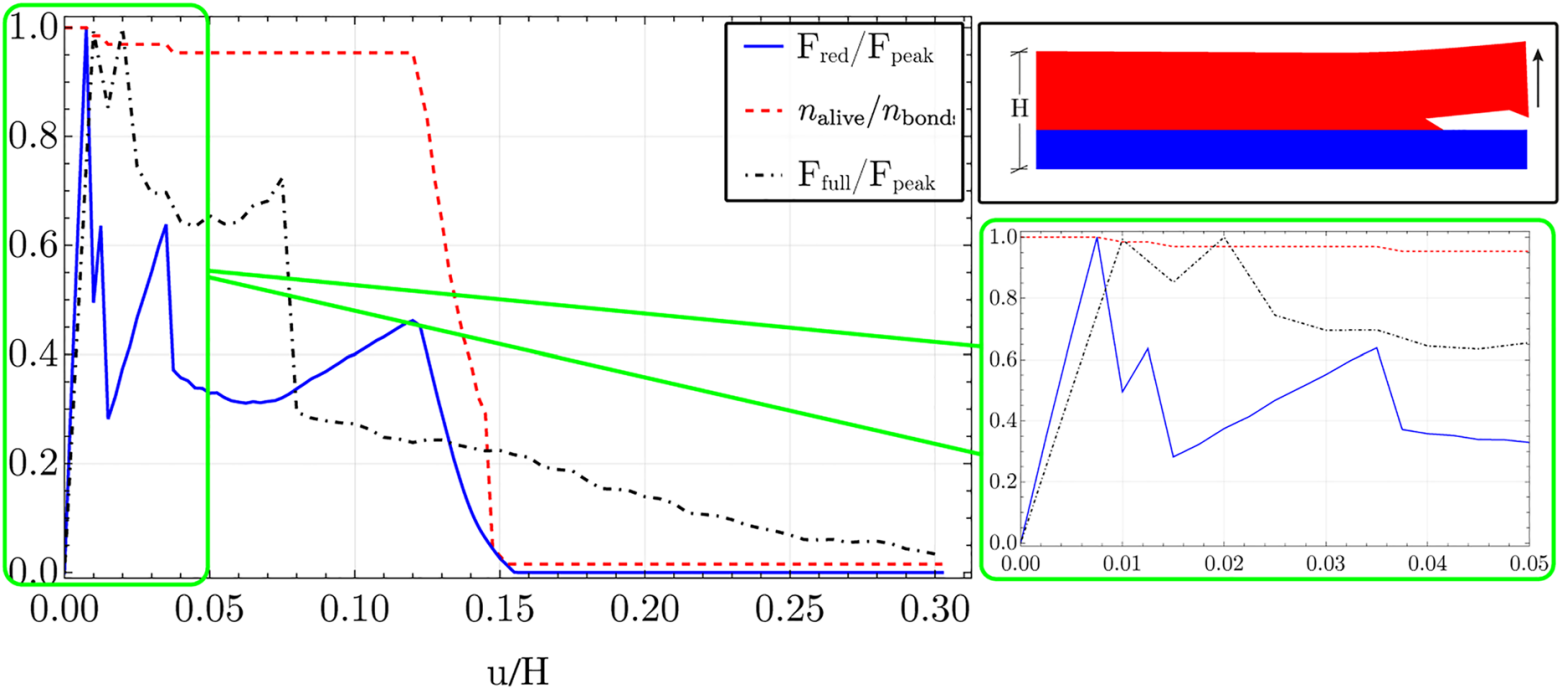
Results of the peeling simulations carried out on a thin layer of a nonlocal microstructure using the reduced formulation presented above (continue line in blue) and a full dimensional model (dot-dashed line in black), expressed in terms of force (normalised with respect to peak force of the fully-dimensional formulation) versus displacements (normalised to specimen height H). In red is the ratio between intact and total bonds, representing effective damage nucleation and propagation

Force versus displacement plots, Fig. 7 shows a qualitative correspondence between the dimensionally reduced peridynamic formulation and the full dimensional discrete approximation. Peaks occur at similar values of the force but at slightly different values of the imposed displacement. It is believed that this difference is due to the approximation introduced by the ansatz assumed for the displacement field while obtaining the dimensionally reduced formulation. Indeed, for the case portrayed in the various figures of the present section, only first-order terms in the through-thickness coordinate have been considered for the absolutely continuous part of the displacement. Despite this clear limitation, improvements in the stiffness of the structure could be achieved by assuming such a displacement field with higher-order terms in the through-thickness variable. Nonetheless, a generally good qualitative and quantitative agreement is found between the two modelling approaches.

### Reducing the nonlocal range of interaction

 We here explore the effects of reducing the nonlocal feature of the upper layer (Material 1 of Fig. 3) on its failure mechanism.

**Fig. 8:**
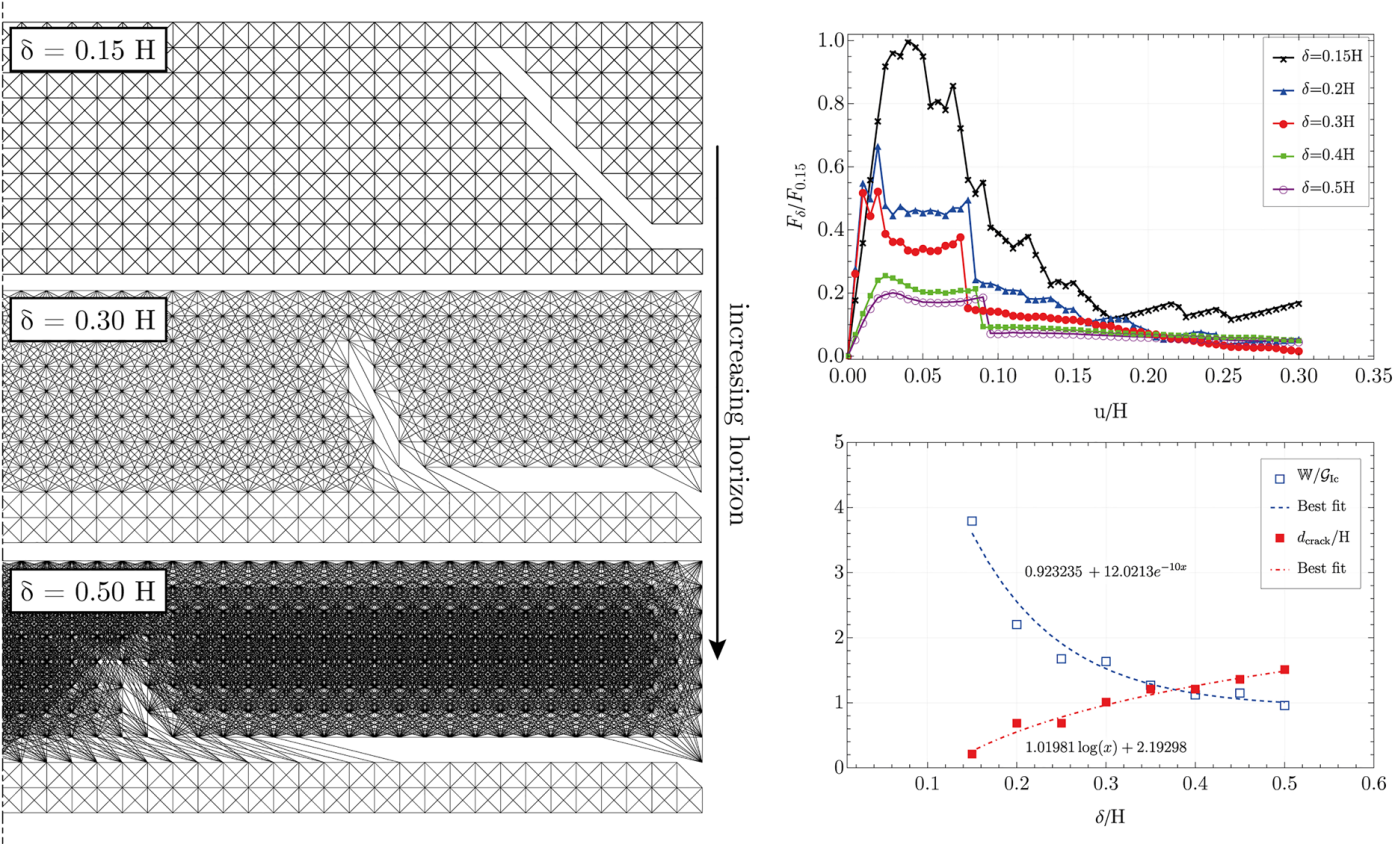
On the left: side view of *post-mortem* specimen subjected to peeling test. The specimen’s upper layers are obtained with an increasing nonlocal character ($\delta$), whereas the lower layers behave locally. On the right upper part: force versus displacement plots (normalised with respect to the peak force registered among all the different cases) resulting from the peeling test of thin films with varying nonlocal horizons $\delta$. On the lower right: for various ratios of horizons to thin element height ($\delta /$H), the ratio between the work done to deform the structure at the end of the test and surface free energy ($\mathbb {W}/\mathcal {G}_{Ic}$) and the length of the crack to plate height ratio ($d_{crack }/H$), are reported

In particular, nonlocality has the role of promoting horizontal crack propagation delaying the growth of damage inside the upper layer until a distance well beyond the height of the whole element. In this sense, damage to the thin upper layer is located at a distal section from the loaded end. This effect is captured in Fig. 8 through a sensitivity analysis of the effect of the horizon length on crack nucleation and propagation. In particular, Fig. 8, summarises these findings by showing (on the left) three specimens with different nonlocal lattice networks for the upper layer—which are the discrete equivalent of bond-based peridynamics—and the path taken by the crack at the end of the peeling test. It is quite clear how even in the conditions of constant stiffness and strength of the composite, a nonlocal microstructure can foster different failure behaviours and lead to the formation of a delamination surface running at the interface with the substrate. Furthermore, an increasing nonlocal trait corresponds to a change in the orientation of the crack path when it deviates from the interface into the upper material. Indeed, the path tends to become more and more vertical with the growing horizon length. The right side of Fig. 8 shows the force versus displacement plots of the above-mentioned peeling test carried out on several nonlocal materials. All of the numerical simulations are carried out by keeping an equal overall stiffness and strength but different horizon lengths, i.e. nonlocal interaction.

From the force versus displacement plot (Fig. 8 on the right), it emerges that lower horizons lead to stronger structures storing more energy in the form of straining recoverable work, although that leads to failure. This is characterised by smaller internal surfaces (Fig. 8 on the right) that, as seen from the previous paragraphs, evolve immediately in the upper layer and stay limited to a region very close to the loaded end. Figure 8 at its lower right corner displays the ratio between the external work done by lifting the free edge (i.e. the input energy), the surface free energy (reported with the blue empty squares), and the ratio between the length of the free surface created in the solid and the element thickness H. The latter is a quantity proportional to the dissipated energy. Therefore, to greater distance between the blue dashed curve and the red dashed one, corresponds to a greater quantity of strain energy stored in the structure.

Larger horizons, on the contrary, make the structure more and more compliant at the borders. Such an inhomogeneity ends up attracting the crack path that ultimately leads to smaller forces required to produce crack propagation. The more the microstructure is nonlocal the more the propagation travels horizontally to greater distances (see Fig. 8 on the left), allowing the damage to the upper layer to grow in a distal region of the solid.

## Conclusions

In the present work, a dimensionally reduced and fully computational formulation of peridynamic bond-based thin structures in the nonlinear regime is obtained. Theoretical aspects related to the nonlinear generalisation of the dimensionally reduced perydinamic model are object of ongoing efforts and possible future works. The model stems from a previously published work of the Authors that treated the very onset of early-stage fracture nucleation and growth. Crack propagation until complete specimen failure is instead explored in this present work by employing a Hencky-type pairwise potential function. This is to account for geometric nonlinearities. The proposed model is used here to address a specific case study. That consists of a thin layer of nonlocal material perfectly bonded on top of a soft substrate and subject to a displacement-induced peeling test. The results from the model are compared with a fully three-dimensional analysis (conducted on the same case study) by implementing a discrete approximation of bond-based peridynamics in ANSYS^®^. It is found that the proposed reduced formulation gives a good qualitative and quantitative agreement with the numerical results obtained with ANSYS^®^, even though a perfect match between those two solutions is not yet achieved, primarily because a first-order approximation of the through-thickness displacements is utilised. From a sensitivity analysis performed on both models (dimensionally reduced and fully discrete), a strong dependence of the crack growth on the horizon (describing the degree of nonlocality) is assessed.

It is found that a pronounced nonlocality of the upper peeled layer can lead to a decrease in the force required for peeling failure. At the same time, a more local feature is associated with a destructive fracture process, in which the crack propagates in the vicinity of the loaded section and the upper material. Given the equivalence between a nonlocal continuum and its associated lattice network (valid for the bond-based peridynamic model), it is seemingly appropriate to speak about nonlocal microstructures for the cases at hand. Such submacroscopic structures thus show a crack path propagating at the boundary between the two materials and then deviating in the upper layer. This occurs only at a later stage, and it causes damage to portions of the body which are far away from the loaded end.

All of the above-mentioned aspects can have various implications in the engineering field. In particular, the fact that certain microstructures reduce the force required for peeling implies a more efficient and possibly controlled peeling process. This may have implications for practical applications where minimising the required force is critical, such as in the design of adhesive joints or protective coatings. Furthermore, a less destructive failure mode suggests a more controlled and predictable response during peeling. This is advantageous in scenarios where keeping the structural integrity of the material is crucial. Applications in industries like aerospace, automotive, or electronics, where materials often face extreme loading conditions, could benefit from this enhanced failure mode. Microstructure manipulation as a tool for optimising the material behaviour in peeling scenarios opens up avenues for designing materials with customised and specific mechanical characteristics, leading to different application requirements. Understanding how microstructures influence peeling forces can be particularly valuable in the design of adhesive joints. The ability to reduce peeling forces while ensuring a less destructive failure mode could lead to improved performance and durability of bonded structures. Lastly, lowering the force required for peeling has potential implications for energy efficiency. Processes involving peeling, such as manufacturing or packaging, could benefit from reduced energy consumption, contributing to sustainability goals. The findings presented in this work could inspire further research into different types of microstructures and their effects on peeling behaviour. This could yield a more comprehensive understanding of the relationship between microstructural design and mechanical properties affecting peeling.

## Data Availability

Not applicable.

## Appedix A: A network of nonlinear trusses

The aim of this section is to obtain the expression of the pairwise potential function, $\omega$, and pairwise force function, $\varvec{f}$, for a bond-based peridynamic continuum accounting for large deformations. In bond-based peridynamics, the bond connecting pairs of material particles corresponds to a truss, as it exchanges forces aligned along the direction connecting the pairs. It is assumed here, for each bond a nonlinear constitutive relation resulting from a one-dimensional application of Hencky’s theory of deformation. In the three-dimensional case, Hencky’s strain energy density [83] can be written as

A1 $$\begin{aligned} \psi =\frac{\Lambda }{2}(tr \varvec{h})^2+G\ \varvec{h}:\varvec{h}\quad , \end{aligned}$$

where $\varvec{h}=\log \varvec{V} = \log \lambda _i\ \varvec{e}_i\otimes \varvec{e}_i$ is the logarithmic strain tensor, $\varvec{V}$ being the left stretch tensor [81], while $\Lambda$ and *G* are the two independent constants—called Lamé constants—that specify the unique isotropic response of the material to which the energy (A1) is associated. In a uniaxial loading framework, a truss is subjected to a particular deformation regime, namely by assuming $\lambda _1$ to be the stretch in the longitudinal direction and $\lambda _{2,3}$ the stretches in the transversal directions:

A2 $$\begin{aligned} \frac{\partial \psi }{\partial \lambda _2}=P_{22}=0\quad , \quad \frac{\partial \psi }{\partial \lambda _3}=P_{33}=0\quad . \end{aligned}$$

Here, the $P_{ij}$s are the components of the first Piola–Kirchhoff stress tensor. Enforcing conditions (A2) onto Eq. (A1) leads to the following strain energy density:

$$\begin{aligned} \psi =\frac{G(2G+3\Lambda )}{2(G+\Lambda )}(\log \lambda _1)^2=\frac{E}{2}(\log \lambda )^2\quad , \end{aligned}$$

where the last equality is obtained by labelling $\lambda _1=\lambda$ and introducing the relation between the Lamé constants and Young’s Modulus *E*. Thus, the total elastic energy stored in a truss of cross-sectional area at rest A and total length at rest $|\varvec{\xi }|$, can be written as

A3 $$\begin{aligned} \Psi = \psi \ A \ |\varvec{\xi }| = \frac{1}{2}EA \ |\varvec{\xi }| (\log \lambda )^2\quad . \end{aligned}$$

It is possible now to compare the energy of (A3) with that emerging from the peridynamic bond-based interpretation of a continuum in order to determine its formulation. In fact, by considering two very small interacting regions *V* and $V'$ (representing volumes in 3D and areas in 2D) having $\textbf{X}$ and $\textbf{Y}$ as two internal points of *V* and $V'$ respectively, the total energy due to any type of elastic deformation can be evaluated as:

A4 $$\begin{aligned} \Psi =\int _V\int _{V'} \omega (\textbf{x},\textbf{y})\textrm{d}\textbf{x}\textrm{d}\textbf{y} = \omega (\textbf{X},\textbf{Y})\ V\ V'\quad , \end{aligned}$$

where $\omega$ is the pairwise peridynamic potential function, and the last equality is in virtue of the mean value theorem.

Equating Eqs. (A3) and (A4) leads to the identification of the pairwise potential function for bond-based peridynamics accounting for Hencky’s strain measures:

A5 $$\begin{aligned} \omega = \frac{1}{2}\frac{EA}{V\ V'} |\varvec{\xi }|(\log \lambda )^2\quad ; \end{aligned}$$

here a dependence on $\textbf{X}$ and $\textbf{Y}$ is to be intended for the quantities *V*, $V'$, $\varvec{\xi }$ and $\lambda$. It is now possible to derive the “Hencky” pairwise force density as

$$\begin{aligned} \varvec{f}_H=\frac{\partial \omega }{\partial \varvec{\eta }}=\frac{\partial \omega }{\partial \lambda }\frac{\partial \lambda }{\partial \varvec{\eta }}=\frac{EA}{V\ V'}\frac{\log \lambda }{\lambda } \varvec{e}^*\quad , \end{aligned}$$

which by direct comparison with the bond-based pairwise force function $\varvec{f}=c\ s\ \varvec{e}^*$ leads to:

A6 $$\begin{aligned} c=\frac{EA}{V\ V'}\quad , \quad s(\varvec{\eta })=\frac{\log \lambda }{\lambda }\quad . \end{aligned}$$

## Code availability

The code utilised for generating the presented results is available from the authors upon request.
